# Efficacy of esketamine on perioperative anxiety in patients receiving anesthesia: a systematic review and meta-analysis of RCTs

**DOI:** 10.3389/fpsyt.2026.1721985

**Published:** 2026-02-03

**Authors:** Shang Shi, Feng Wang, Jieqiong Luo, Fangzhou Yang, Xiaohua Zou

**Affiliations:** Department of Anesthesiology, The Affiliated Hospital of Guizhou Medical University, Guiyang, Guizhou, China

**Keywords:** anxiety, depression, esketamine, meta-analysis, randomized controlled trials

## Abstract

**Objective:**

This study aimed to assess the impact of esketamine on perioperative anxiety, including its frequency, intensity, and score variations, across different surgical procedures by analyzing data from randomized controlled trials (RCTs).

**Methods:**

A comprehensive literature search was performed across PubMed, Embase, the Cochrane Library, and Web of Science to identify relevant RCTs evaluating esketamine. Statistical analyses were conducted using Review Manager version 5.4 and Stata. Studies were included if they were RCTs involving anesthetized adult patients comparing esketamine with others, reporting of perioperative outcomes and sufficient data for meta-analysis. Studies were excluded for being reviews/editorials/case reports, conference abstracts, pediatric-focused, unpublished, or non-English. Primary outcomes contained change in anxiety/depression score and anxiety/depression Score. Secondary outcomes contained change in sleep/pain score and perioperation data. To assess publication bias, funnel plot visualization and Egger’s test were employed.

**Results:**

A total of eight RCTs involving 1,101 participants fulfilled the inclusion criteria. The pooled analysis indicated that esketamine administration led to a significant reduction in perioperative anxiety levels compared to control interventions (SMD = −0.36; 95% CI: −0.67 to −0.06; p < 0.0001; I² = 84%). However, no statistically significant improvements were observed in sleep parameters, pain relief, surgical or anesthetic duration, or intraoperative metrics such as fluid administration, blood loss, urine output, or adverse event rates. Sensitivity analyses suggested variability in the anxiety-related outcomes, while findings related to depression remained consistent. Subgroup evaluations indicated a lack of measurable benefit in individuals under 40 years of age, those undergoing abdominal operations, and patients receiving spinal anesthesia.

**Conclusion:**

Esketamine shows promise in reducing anxiety during the perioperative period. Nonetheless, its effectiveness may depend on individual patient profiles and surgical settings. Further high-quality trials are needed to identify the most effective dosing regimens, delivery methods, and combination strategies to enhance efficacy and reduce side effects.

**Systematic Review Registration:**

https://www.crd.york.ac.uk/prospero/, PROSPERO identifier CRD420251050362.

## 1 Introduction

Anxiety experienced around the time of surgery represents a major psychological burden, provoking a combination of emotional, mental, and physical responses in patients undergoing surgical procedures (1). Globally, the reported prevalence of perioperative anxiety varies considerably, with estimates ranging from 21% to 80% and an overall average prevalence of approximately 48% (2). Anxiety during the perioperative period can arise from worries about potential surgical complications, unfavorable outcomes, loss of autonomy under general anesthesia, or anticipated discomfort such as pain and postoperative nausea and vomiting (2, 3). This type of anxiety is driven by both mental and physiological factors. Heightened sympathetic nervous system activity can result in increased secretion of cortisol, as well as elevated heart rate and blood pressure, potentially disrupting the initiation and stability of anesthesia (4, 5). An intensified physiological stress response has been associated with a range of negative postoperative outcomes, such as nausea, vomiting, pain, sleep disturbances, cognitive decline, slower recovery, extended hospital stays, and a higher risk of long-term mortality (6).

Management of perioperative anxiety involves both drug-based and non-drug interventions. Among pharmacological options, benzodiazepines-especially midazolam-are commonly used because of their fast-acting anxiolytic properties (7). Beta-adrenergic antagonists, such as propranolol, have proven useful in alleviating the physical manifestations of anxiety, including elevated heart rate and high blood pressure (8, 9). In recent years, agents like melatonin and dexmedetomidine have gained attention as promising substitutes for benzodiazepines in the treatment of perioperative anxiety (10, 11). A prospective, randomized, double-blind study demonstrated that administering dexmedetomidine as a premedication can improve sedation before surgery and lessen the stress response triggered by intubation in adult patients.

Several non-drug interventions have proven effective in managing perioperative anxiety. These include cognitive behavioral therapy (CBT), lavender-based aromatherapy, massage, acupuncture, music-based interventions, virtual reality (VR) experiences, and comprehensive pre-surgical counseling. Such methods offer meaningful anxiety reduction without the potential side effects associated with pharmacologic therapies (12).

As the S-enantiomer of ketamine, esketamine binds more strongly to NMDA receptors, achieves therapeutic benefits at reduced doses, and is associated with a lower incidence of side effects compared to the racemic mixture (13). As the only intravenous anesthetic with analgesic properties, esketamine can be safely used for induction and maintenance of general anesthesia at a recommended dose of 0.5–1 mg/kg (14). In opioid−free protocols, anesthesia induction for elective or emergency surgery with esketamine 1–1.5 mg/kg, combined with a maintenance infusion of 1–3 mg·kg^-1^·h^-1^ alongside other sedative agents, can also achieve adequate anesthetic depth (15).

Recent evidence indicates that esketamine may alleviate anxiety-like behavior in animal models, potentially through its regulatory effects on the GABAergic system within the prefrontal cortex (16). Additionally, administering sub-anesthetic doses of esketamine(0.3 mg/kg IV after intubation)during surgery has been reported to lower perioperative anxiety levels in patients undergoing procedures such as breast or thyroid surgery (17), and elective cesarean deliveries (18). Earlier meta-analyses and systematic reviews have primarily focused on the role of esketamine in managing depression during the perioperative period. In a study conducted by Yu Qi, esketamine was shown to significantly decrease both the frequency and intensity of depressive symptoms following surgery (19). Despite growing interest, there is a lack of meta-analyses specifically evaluating the effectiveness of esketamine in managing anxiety. This study sought to perform a systematic review and meta-analysis of randomized controlled trials (RCTs) to assess esketamine’s impact on perioperative anxiety. Additionally, we examined how varying dosages and types of surgical procedures might influence anxiety-related outcomes.

## 2 Materials and methods

### 2.1 Literature search

This analysis was conducted in accordance with the PRISMA 2020 reporting standards (20) and was prospectively registered in the PROSPERO database (registration number: CRD420251050362). PubMed, Embase, and Web of Science were systematically searched up to January 2025 to identify English-language studies that evaluated the effectiveness of esketamine in alleviating perioperative anxiety among patients receiving anesthesia. The database search was performed using a combination of the following keywords and terms: “esketamine”, “S-Ketamine”, “Anxiety”, “Angst”, “Nervousness”, “Hypervigilance”, “Social Anxiety”, “Social Anxieties “and “Anxiousness”. **Supplementary Table S1** provides the full search strategy. In addition, the reference lists of all included studies were manually reviewed. Two reviewers independently conducted the literature screening and study selection, resolving any disagreements through discussion until consensus was reached. Eligible studies were selected based on the following criteria: (1) randomized controlled trials; (2) inclusion of adult patients receiving anesthesia; (3) comparison between esketamine and other agents, such as saline or sufentanil; (4) reporting of at least one perioperative outcome-such as anxiety, depression, pain, recovery metrics, sleep quality, duration of surgery or anesthesia, PACU time, extubation time, medication usage, patient satisfaction, blood loss, fluid balance, urine output, length of hospital stay, or postoperative complications; and (5) availability of sufficient data to compute either risk ratios (RRs) or standardized mean differences (SMDs). Studies were excluded if they were reviews, editorials, letters, case reports, conference abstracts, pediatric-focused, unpublished, or written in languages other than English.

### 2.2 Data extraction

Data extraction was conducted independently by two reviewers (Shang Shi and Jieqiong Luo), and any inconsistencies were resolved through consultation with a third researcher (Feng Wang). The collected data included details such as the first author, year of publication, duration of the study, geographic location, research design, number of participants, patient age, body mass index (BMI), American Society of Anesthesiologists (ASA) classification, and perioperative outcomes. These outcomes encompassed measures of anxiety, depression, pain, recovery, sleep quality, surgical and anesthesia duration, PACU and extubation times, drug usage, patient-reported satisfaction, intraoperative blood loss, fluid management, urinary output, length of hospitalization, and any recorded complications. When studies presented continuous variables as medians with ranges or interquartile ranges, the mean and standard deviation were calculated using established statistical conversion methods. In cases where essential data were missing or not disclosed, attempts were made to contact the corresponding authors to retrieve the required information.

### 2.3 Quality assessment

The methodological quality of the included randomized controlled trials (RCTs) was assessed based on the criteria outlined in the Cochrane Handbook for Systematic Reviews of Interventions (version 5.1.0) (21). The evaluation covered seven key domains: generation of the randomization sequence, concealment of allocation, blinding of both participants and study personnel, blinding of outcome assessors, handling of incomplete outcome data, reporting bias, and any other potential methodological concerns.

### 2.4 Statistical analysis

Data synthesis was carried out using Review Manager (RevMan) version 5.4, developed by the Cochrane Collaboration (Oxford, UK). For continuous variables, SMDs were applied, while dichotomous outcomes were analyzed using risk ratios (RRs). All effect sizes were presented with 95% confidence intervals (CIs). To assess statistical heterogeneity, the chi-squared test (Cochran’s Q) was employed and further quantified using the I² statistic. Heterogeneity was considered statistically significant when the p-value was less than 0.05 or the I² exceeded 50%. When such heterogeneity was present, a random-effects model was used to calculate the combined effect estimates. Sensitivity analyses using a one-way approach were conducted to test the stability of the findings, while subgroup analyses were performed to identify possible sources of variability. To evaluate potential publication bias, funnel plots were visually inspected using RevMan 5.4, and Egger’s regression test was applied in Stata version 18.0 (StataCorp, College Station, TX, USA) for outcomes reported in ten or more studies. A p-value below 0.05 was considered indicative of publication bias.

## 3 Results

### 3.1 Literature search and study characteristics

**Figure 1:**
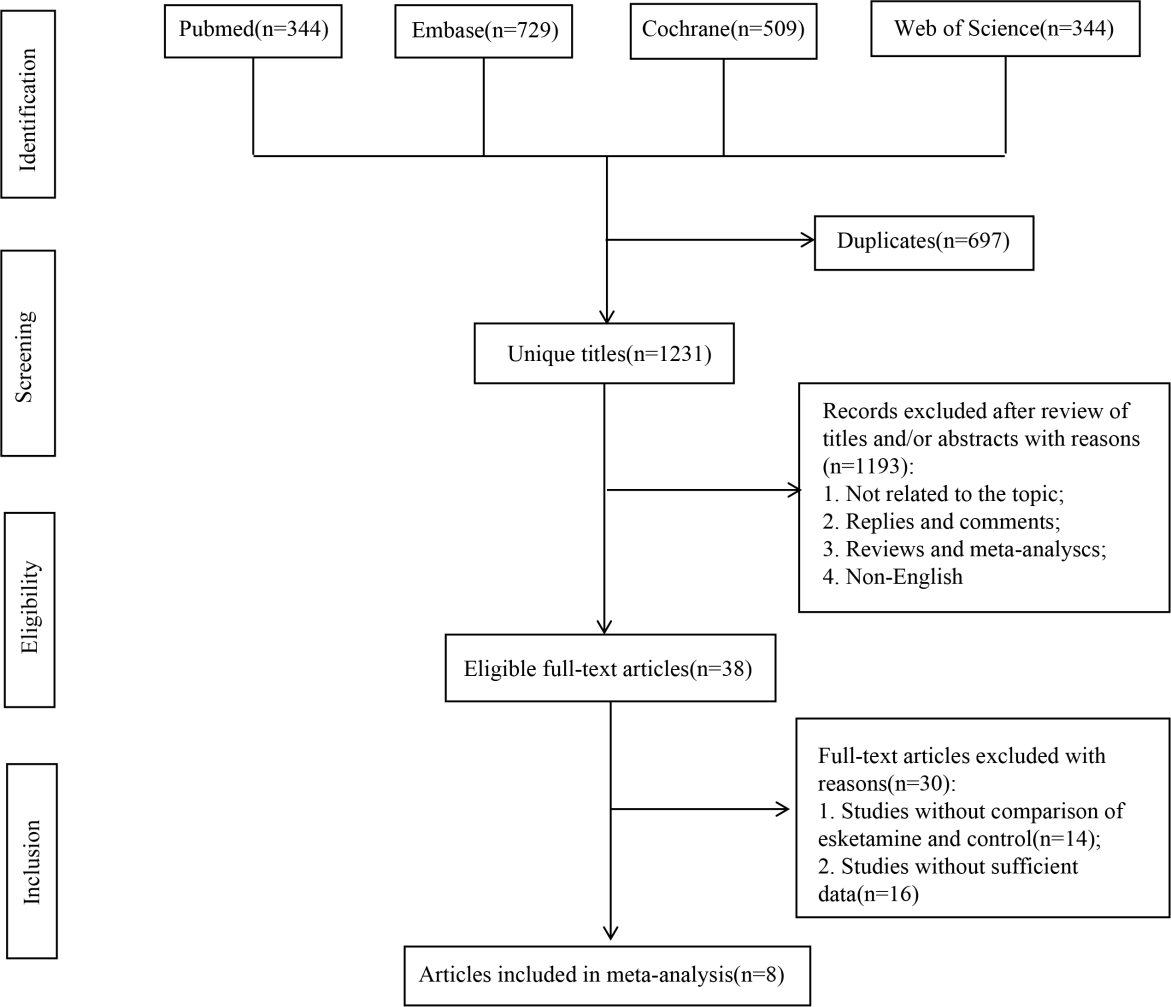
Flowchart of the systematic search and selection process.

The process of study selection is illustrated in **Figure 1**. A total of 1,928 records were retrieved from four databases: PubMed (n = 344), Embase (n = 729), the Cochrane Library (n = 509), and Web of Science (n = 344). Following the elimination of duplicate entries, 1,231 titles and abstracts were screened. Ultimately, 8 randomized controlled trials (17, 18, 22–27) comprising 1,101 participants-553 assigned to the esketamine group and 548 to the control group-were included in the final meta-analysis. All studies met the criteria of randomized controlled design. Key characteristics, such as type of anesthesia, nature of surgical procedures, demographic data (age, sex), BMI, and American Society of ASA classification, are summarized in **Table 1**. A comprehensive evaluation of study quality is provided in **Supplementary Figure S1**.

**Table 1:** Demographics and clinical characteristics of included studies.

Author	Study period	Region	Study design	Registration number	Anesthesia		Surgery	Intervention	Control	The method of anxiety assessment	The time of anxiety assessment
Xiang Cheng et al.	2021	China	RCT	ChiCTR2100046552	general anesthesia		Video-Assisted Thoracic Surgery	esketamine,0.25 mg/kg, followed by an infusion of 0.125 mg/kg/h until 15 minutes before the end of the surgical procedure(n=39)	saline, identical volumes and rates of 0.9% saline(n=38)	HADS-A	POD 2
Di Qiu et al.	2021-2022	China	RCT	ChiCTR2100048587	general anesthesia		gynecological laparoscopic surgery.	esketamine, 0.3 mg/kg/h(n=92)	saline(n=91)	HADS-A	POD 1;POD 3
Lili Yu et al.	2022	China	RCT	ChiCTR2200056391	general anesthesia		elective MRM for unilateral breast cancer (UBC)	esketamine,0.25 mg/kg(n=68)	sufentanil,0.25 μg/kg(n=68)	HADS-A	Hospital discharge
Shu-lin Gan et al.	2021-2022	China	RCT	ChiCTR2100046194	general anesthesia		thoracoscopic lung cancer surgery	Esketamine,0.1 mg/kg was administered intravenously at about 3 min before skin incision, followed by a continuous infusion of 0.1 mg/kg/h until the end of surgery(n=78)	saline group, the same volume(n=78)	BAI	POD 2;hospital discharge
Dongxu Zhou et al.	2021-2022	China	RCT	ChiCTR2200060928	general anesthesia		breast or thyroid surgery	esketamine,0.3 mg/kg(n=60)	saline, an equal volume of normal saline(n=60)	SAS	POD 1;POD 2;POD 3
Rong Zhou et al.	2021	China	RCT	ChiCTR2100042140	general anesthesia		elective Video-Assisted thoracoscopic surgical	esketamine,0.5 mg/kg(n=195)	normal saline(n=192)	HADS-A	POD 1;POD 3
Haotian Chen et al.	2023-2024	China	RCT	ChiCTR2400080363	intravenous sedation		liposuction surgery	esketamine,0.15–0.3 mg/kg/h(n=78)	saline, an equivalent volume of saline(n=77)	HADS-A	POD 1;POD 3
Yu Qi et al.	2022-2023	China	RCT	ChiCTR2300078343	spinal anesthesia		elective cesarean sections	esketamine group,0.2 mg/kg(n=60)	saline group, the same volume(n=60)	STAI-S	POD 1;POD 7
Author	Mean/median Age			Male/female		Mean/median BMI			Mean/median ASA score		
	Esketamine		Control	Esketamine	Control	Esketamine		Control	Esketamine		Control
Xiang Cheng et al.	58.09 ± 9.04		55.80 ± 7.51	16/23	14/24	weight 66.20 ± 9.90		67.50 ± 11.40	2-3(32/7)		2-3(33/5)
Di Qiu et al.	41.24 ± 12.80		42.89 ± 10.54	0/92	0/91	23.48 ± 3.62		23.91 ± 3.09	1-3(58/32/2)		1-3(54/35/2)
Lili Yu et al.	46.00 ± 10.00		48.00 ± 9.00	0/68	0/68	22.40 ± 1.60		22.10 ± 2.00	1-2(56/13)		1-2(52/17)
Shu-lin Gan et al.	54.00 ± 11.70		53.80 ± 12.10	14/54	8/60	23.10 ± 2.70		23.10 ± 2.60	1-3(35/42/1)		1-3(32/45/1)
Dongxu Zhou et al.	48.30 ± 11.49		47.33 ± 12.12	0/60	0/60	Weight (kg) 60.28 ± 10.20		61.75 ± 10.54	2-3(38/22)		2-3(34/26)
Rong Zhou et al.	52.08 ± 8.77		50.74 ± 9.48	72/123	83/109	23.54 ± 2.75		23.59 ± 2.90	2-3(52/143)		2-3(52/140)
Haotian Chen et al.	33.30 ± 10.10		33.10 ± 10.90	20/58	17/60	22.83 ± 3.70		21.56 ± 2.72	1-2(73/5)		1-2(74/3)
Yu Qi et al.	30.53 ± 3.75		30.28 ± 3.68	0/60	0/60	26.68 ± 1.71		26.94 ± 1.40	1-2(49/11)		1-2(47/13)

HADS-A, Hospital Anxiety and Depression Scale Anxiety subscale; BA, Beck Anxiety Inventory; SAS, Standard deviation; STAI-S, Preoperative State-Trait Anxiety Inventory State scores.

### 3.2 Meta-analysis of primary outcomes

#### 3.2.1 Change in anxiety score

**Figure 2:**
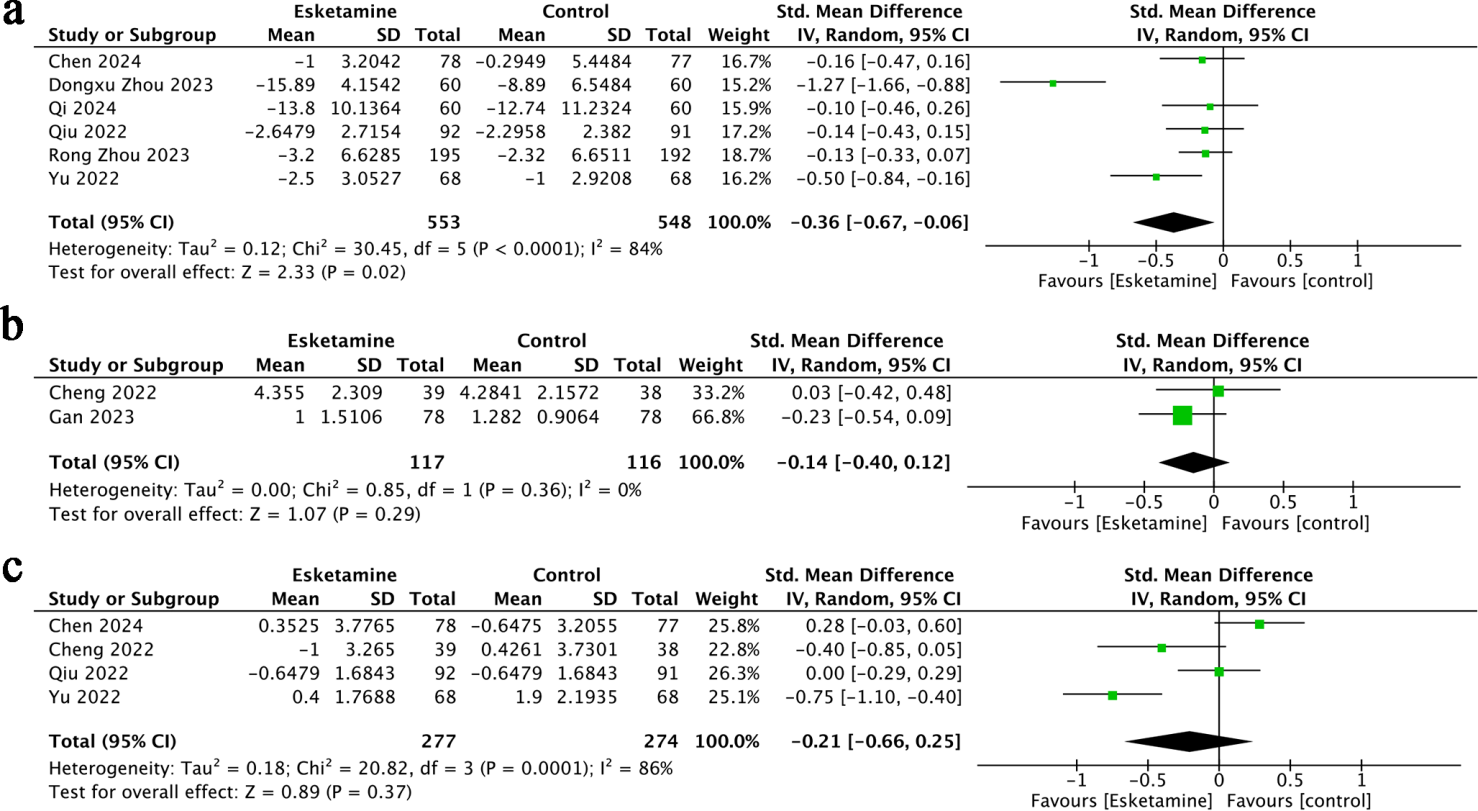
Forest plots of Change in Anxiety Score **(A)**, Anxiety Score **(B)**, Change in Depression Score **(C)**.

**Figure 3:**
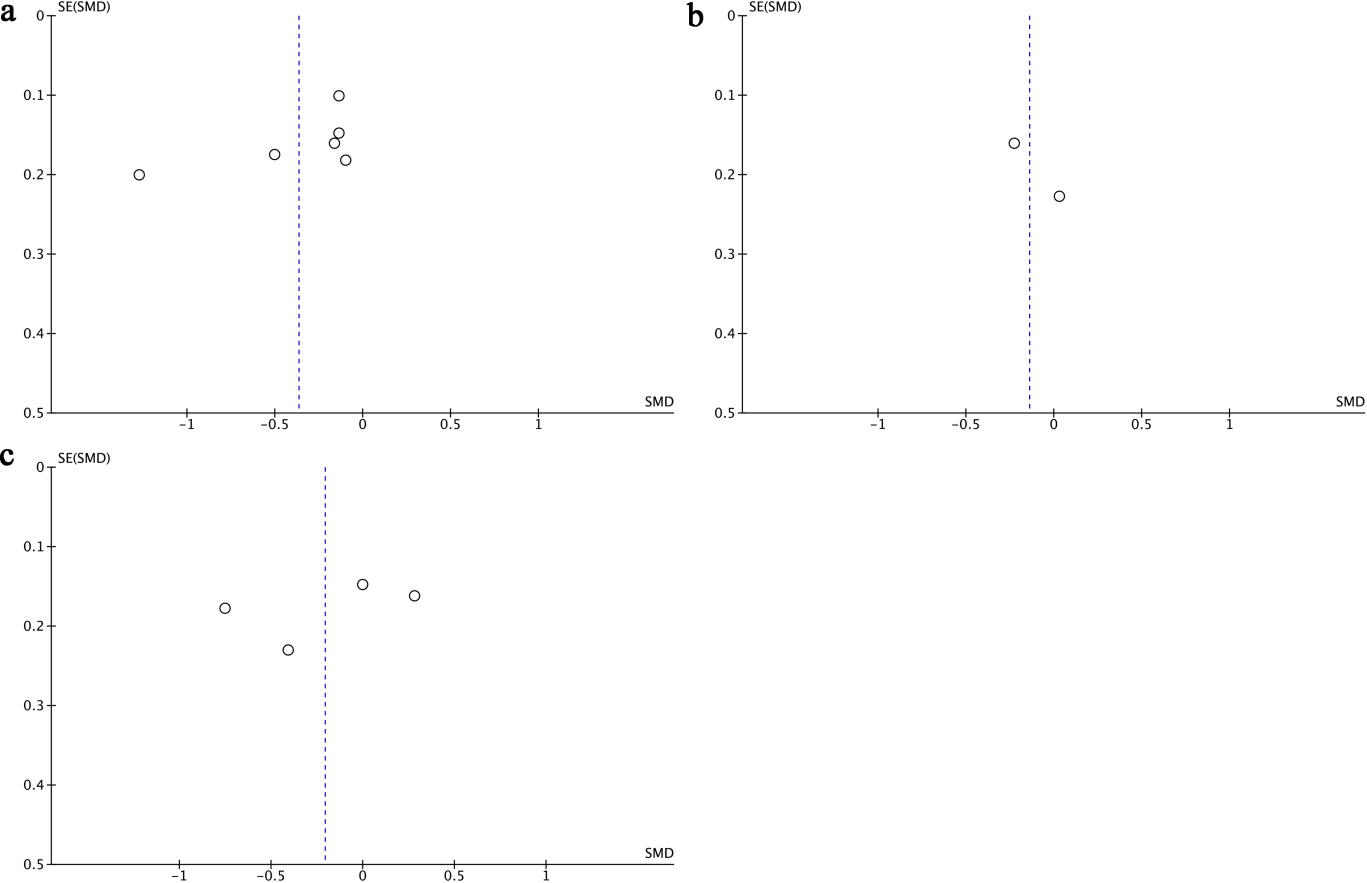
Funnel plots of Change in Anxiety Score **(A)**, Anxiety Score **(B)**, Change in Depression Score **(C)**.

Change in anxiety score refers to the difference between the postoperative anxiety levels in the esketamine or control group and respectively preoperative anxiety levels. Six studies (17, 18, 22, 25–27) provided data on changes in anxiety scores, including a total of 1,101 participants-553 treated with esketamine and 548 in the control group. The combined analysis demonstrated that anxiety scores were significantly lower in the esketamine group compared to controls (SMD = −0.36; 95% CI: −0.67 to −0.06; p < 0.0001). However, considerable heterogeneity was observed among the studies (I² = 84%, p = 0.02) (**Figure 2A**). Visual examination of the funnel plot indicated a mild possibility of publication bias (**Figure 3A**), although Egger’s regression analysis did not reach statistical significance (p = 0.218).

#### 3.2.2 Anxiety score

Two studies (23, 24) reported anxiety score outcomes for a total of 233 patients-117 in the esketamine group and 116 in the control group. The pooled analysis indicated no statistically significant difference between the two groups (SMD = −0.14; 95% CI: −0.40 to 0.12; p = 0.36), and heterogeneity was negligible (I² = 0%, p = 0.29) (**Figure 2B**). Visual inspection of the funnel plot (**Figure 3B**) showed no signs of publication bias.

#### 3.2.3 Change in depression score

Four studies (22, 23, 25, 26) reported data on changes in depression scores, including a total of 551 participants-277 received esketamine and 274 were assigned to the control group. The meta-analysis revealed no statistically significant difference between the two groups (SMD = −0.21; 95% CI: −0.66 to 0.25; p = 0.37), and notable heterogeneity was observed across studies (I² = 86%, p = 0.0001) (**Figure 2C**). While visual assessment of the funnel plot (**Figure 3C**) suggested a minor publication bias, Egger’s test did not confirm statistical significance (p = 0.061).

### 3.3 Secondary outcomes

#### 3.3.1 Change in sleep score

**Figure 4:**
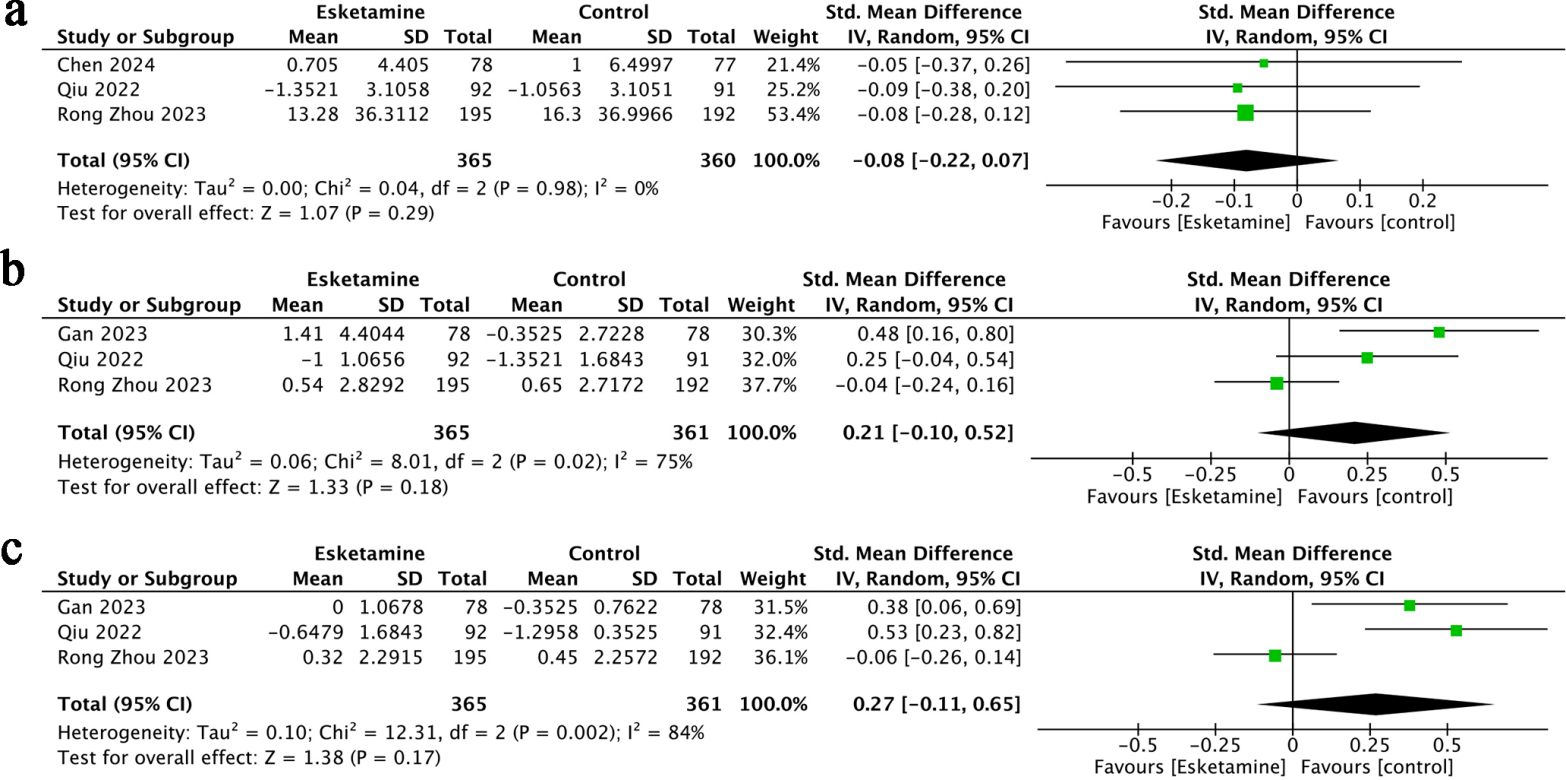
Forest plots of Change in Sleep Score **(A)**, pain score at rest **(B)**,pain score at rest **(C)**.

Sleep quality outcomes were reported in three studies (22, 25, 27), including a total of 725 participants-365 treated with esketamine and 360 in the control group. The combined results indicated no significant difference between the two groups (SMD = −0.08; 95% CI: −0.22 to 0.07; p = 0.98). No heterogeneity was observed among the studies (I² = 0%, p = 0.29) (**Figure 4A**).

#### 3.3.2 Change in pain score

Postoperative pain scores-both at rest and during movement-were reported in three studies (24, 25, 27), involving 726 participants (365 in the esketamine group and 361 in the control group). The pooled results showed no statistically significant differences between the two groups, whether preoperation pain score and postoperation pain score was assessed at rest (SMD = 0.27; 95% CI: −0.11 to 0.65; p = 0.17; I² = 84%) or during movement (SMD = 0.21; 95% CI: −0.10 to 0.52; p = 0.18; I² = 75%) (**Figures 4B, C**).

#### 3.3.3 Perioperation data

**Figure 5:**
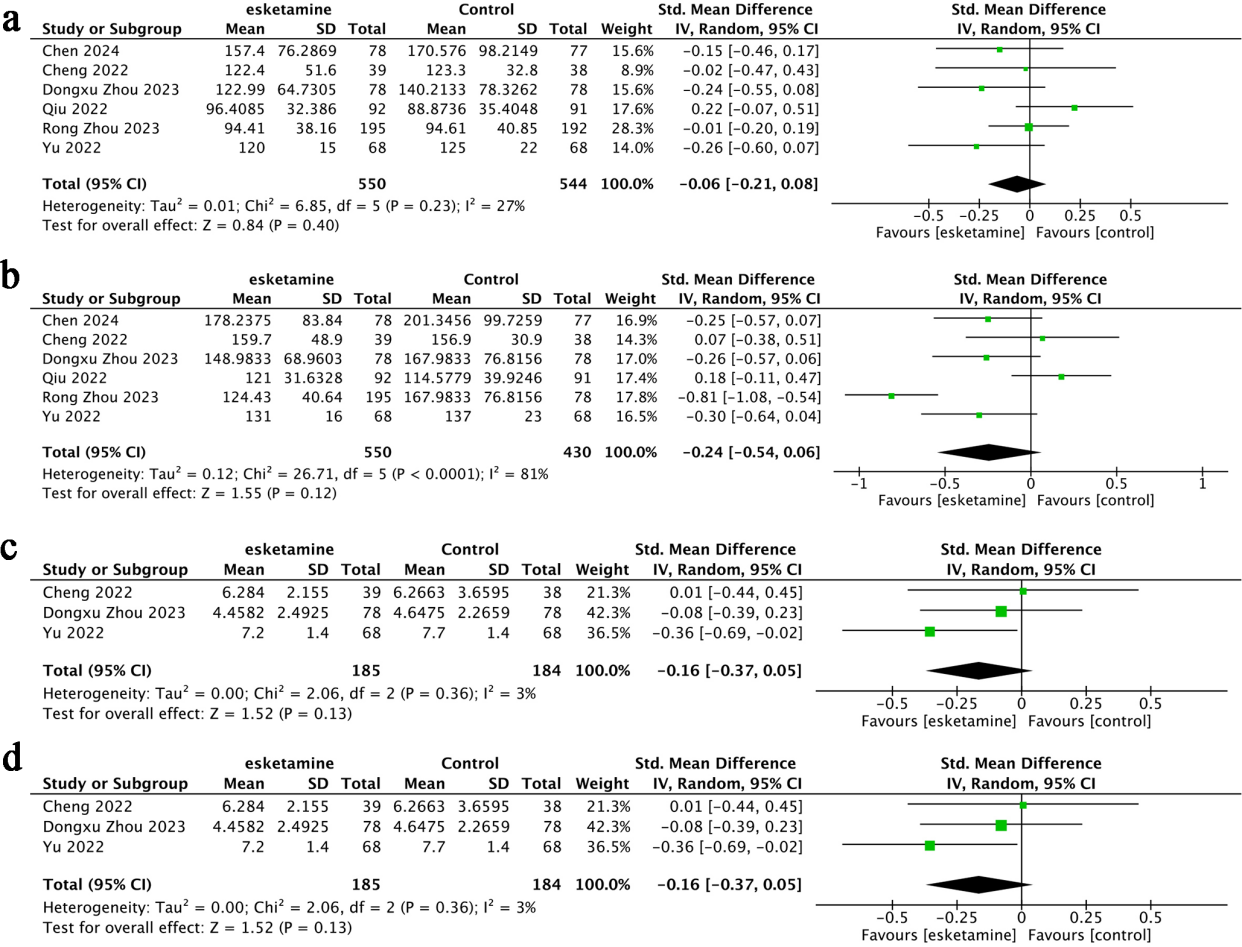
Forest plots of operation time **(A)**, duration of anesthesia **(B)**, time spent in the post-anesthesia care unit (PACU) **(C)**, or length of postoperative hospitalization **(D)**.

**Figure 6:**
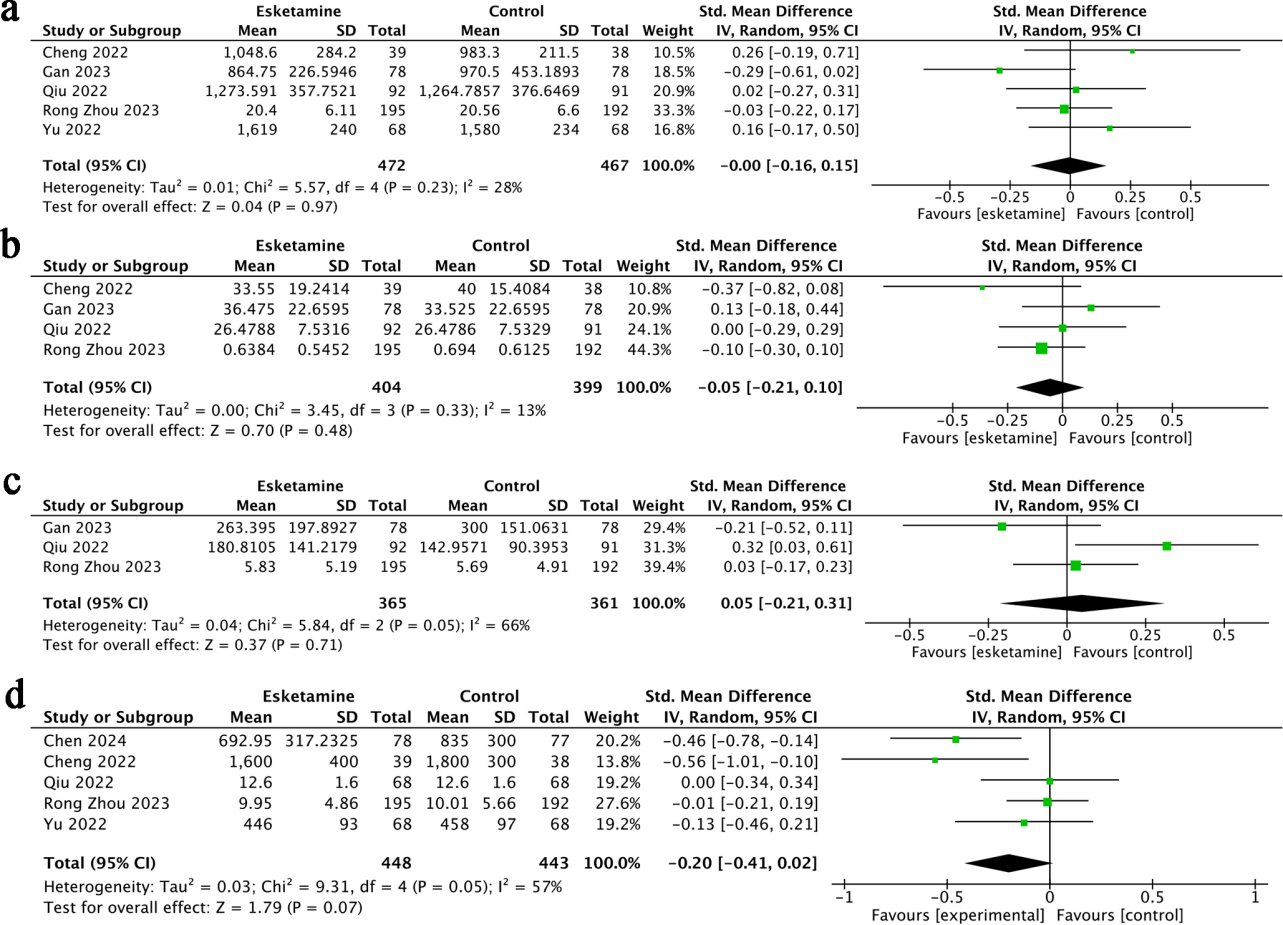
Forest plots of the volume of fluids administered **(A)**, intraoperative blood loss **(B)**, urine output **(C)**, or remifentanil usage **(D)**.

**Figure 7:**
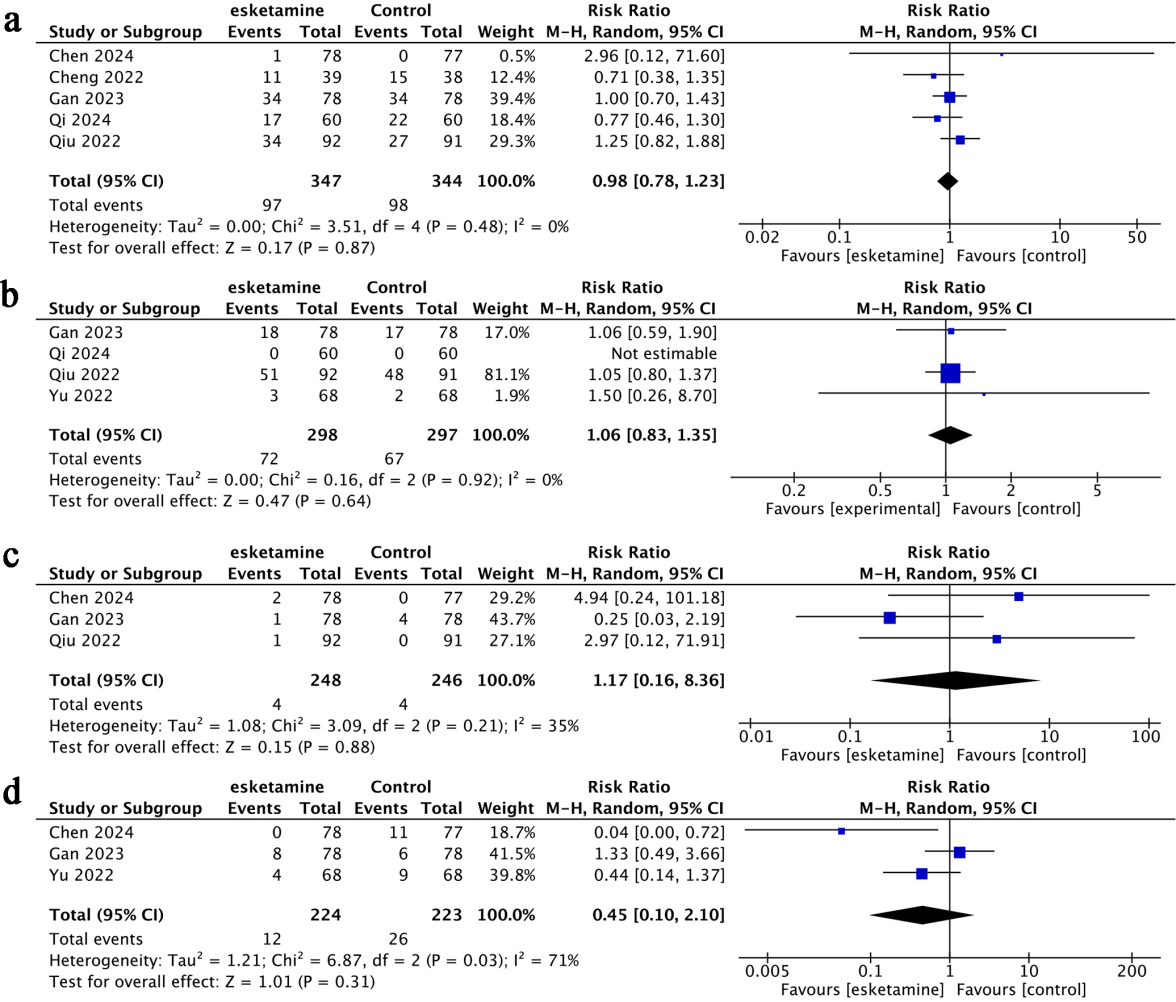
Forest plots of postoperative nausea and vomiting (PONV) **(A)**, dizziness **(B)**, nightmares **(C)**, and bradycardia **(D)**.

Compared to the control group, esketamine did not result in a significant reduction in operation time (**Figure 5A**), duration of anesthesia (**Figure 5B**), time spent in the post-anesthesia care unit (PACU) (**Figure 5C**), or length of postoperative hospitalization (**Figure 5D**). Additionally, no notable differences were found between groups regarding the volume of fluids administered (**Figure 6A**), intraoperative blood loss (**Figure 6B**), urine output (**Figure 6C**), or remifentanil usage (**Figure 6D**). Likewise, the frequency of adverse events-including postoperative nausea and vomiting (PONV) (**Figure 7A**), dizziness (**Figure 7B**), nightmares (**Figure 7C**), and bradycardia (**Figure 7D**)-was similar across both groups.

### 3.4 Sensitivity analysis

**Figure 8:**
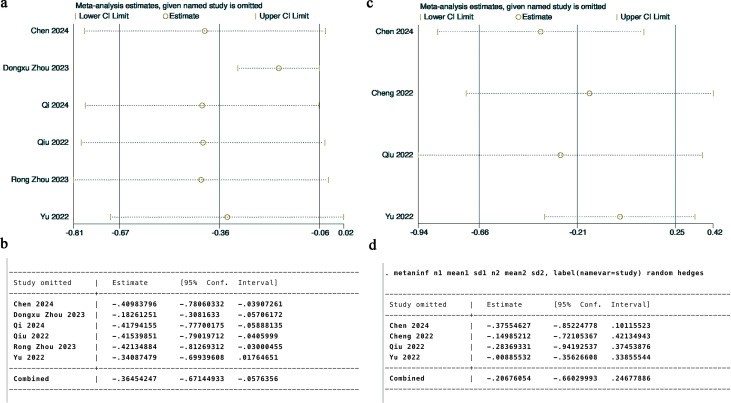
Sensitivity analysis of anxiety outcomes **(A, B)** and depression outcomes **(C, D)**.

One-way sensitivity analyses were conducted for both anxiety and depression outcomes by systematically excluding each study to evaluate its influence on the overall pooled SMDs. The analysis revealed that the study by Yu et al. (26) (2022) introduced variability in the anxiety-related findings (**Figures 8A, B**). Likewise, the depression outcome analysis also demonstrated sensitivity to specific studies (**Figures 8C, D**), suggesting that individual trials had a notable effect on the robustness of the overall results.

### 3.5 Subgroup analysis

Subgroup analyses were conducted to assess anxiety and depression outcomes based on patient age, type of surgery, and anesthesia technique (**Table 2**). Among individuals younger than 40 years, those who had abdominal procedures, and those administered spinal anesthesia, esketamine did not produce a statistically significant improvement-contrary to the general findings. However, in all other subgroups, esketamine demonstrated a favorable effect. Interestingly, no heterogeneity (I² = 0%) was observed within the younger age group and the abdominal surgery cohort, indicating that these subgroups may contribute substantially to the heterogeneity detected in the overall analysis.

**Table 2:** Subgroup analyze.

Subgroup	Change in anxiety score				Change in depression score			
	Comparative group	SMD [95%CI]	*P* value	*I* ^2^	Comparative group	SMD [95%CI]	*P* value	*I* ^2^
Total	6	‘-0.36 [-0.67, -0.06]	<0.0001	86%	4	-0.21 [-0.66, 0.25]	0.0001	86%
Mean/median age								
≥40y	4	-0.49 [-0.94, -0.04]	<0.00001	89%	3	-0.38 [-0.85, 0.10]	0.005	81%
<40y	2	-0.13 [-0.37, 0.11]	0.81	0%	1	0.28 [-0.03, 0.60]	0.08	/
Treatment								
cervical or thoracic surgery	3	-0.62 [-1.25, 0.02]	<0.00001	92%	2	-0.61 [-0.94, -0.27]	0.23	29%
abdominal surgery	3	-0.13 [-0.32, 0.05]	0.97	0%	2	0.13 [-0.14, 0.41]	0.19	41%
Anesthesia								
general anesthesia	5	-0.42 [-0.78, -0.06]	<0.00001	86%				
spinal anesthesia	1	-0.10 [-0.46, 0.26]	0.54	/				

## 4 Discussion

Filling this research void is essential, given the high prevalence of preoperative anxiety among adults (2) and its association with increased risks of postoperative complications (3). Esketamine has regulatory approval for both initiating and sustaining general anesthesia, while its intranasal formulation is utilized in managing treatment-resistant depression (TRD) (28). Recent findings indicate that esketamine can counteract anxiety-like behaviors in animal studies, and clinical data suggest that administering low doses during surgery may effectively alleviate perioperative anxiety (16–18).

This meta-analysis assessed the effectiveness and safety profile of esketamine in managing perioperative anxiety, drawing upon eight rigorously conducted randomized controlled trials. The aggregated findings indicate that esketamine may play a beneficial role in alleviating or preventing anxiety during the perioperative period. In addition to anxiety, four included studies evaluated depressive symptoms. However, contrary to the results reported in an earlier meta-analysis (29), our analysis did not identify any statistically significant differences, suggesting that esketamine might exert little to no impact on depression during the perioperative period. Variations in study selection and inclusion criteria likely contribute to the inconsistent findings. As for sleep quality and pain outcomes, esketamine does not seem to offer significant improvements in sleep parameters or pain relief, either during rest or physical activity. However, the incidence of adverse events-such as postoperative nausea and vomiting (PONV), dizziness, and nightmares-did not increase among patients treated with esketamine compared to controls. However, for adverse events, the confidence intervals are wide, indicating that larger sample studies are needed to clarify their safety profile. Subgroup analysis further indicated that esketamine did not demonstrate significant efficacy in individuals younger than 40 years, those undergoing abdominal procedures, or patients managed with spinal anesthesia. While the use of low-dose intravenous esketamine in conjunction with spinal anesthesia has been shown to lower the incidence of hypotension in women with preoperative anxiety undergoing cesarean delivery, it did not lead to a notable reduction in anxiety scores (18).

Although current evidence points to esketamine’s potential in alleviating perioperative anxiety, inconsistencies among studies highlight the necessity for larger-scale, methodologically uniform clinical trials to confirm its effectiveness. Additionally, the data suggest that its anxiolytic effects may be more pronounced in patients aged over 40. Multiple physiological factors may account for this finding. Adults over the age of 40 commonly exhibit age-associated reductions in hepatic metabolic function and renal elimination capacity (30), which can lead to decreased clearance of esketamine. This, in turn, may result in sustained plasma levels and amplify both its anxiolytic and antidepressant properties. Furthermore, deterioration in the integrity of the blood-brain barrier (BBB) associated with aging may facilitate increased esketamine entry into the central nervous system, potentially boosting its clinical effectiveness in older individuals (31). In the aging population, levels of specific N-methyl-D-aspartate (NMDA) receptor subunits like GluN2B tend to decrease with advancing age (32). Esketamine, acting as an NMDA receptor antagonist, may aid in reestablishing synaptic plasticity by dampening excessive receptor activity. Additionally, it promotes the secretion of brain-derived neurotrophic factor (BDNF), a protein crucial for maintaining neuronal health, regulating mood, and facilitating synaptic signaling (33). The decline in neuronal plasticity associated with aging may amplify the observable therapeutic benefits of esketamine in older adults (32). Moreover, individuals above the age of 40 who undergo surgery are often affected by chronic pain, a factor that could influence and potentially enhance their responsiveness to esketamine treatment (34). The combined analgesic and antidepressant properties of esketamine may exert stronger effects in older adults. In contrast, anxiety in younger individuals is frequently driven by psychosocial stressors, such as occupational pressures, whereas in middle-aged and elderly populations, it tends to be related to underlying neurodegenerative processes. Anxiety arising from such neurobiological alterations may respond more readily to modulation of the glutamatergic system, thereby potentially increasing the clinical effectiveness of esketamine in these patients (35).

Declines in sex hormones such as estrogen and testosterone with advancing age may influence the function of NMDA receptors (36), potentially intensifying the antagonistic action of esketamine. This compound has demonstrated efficacy as an adjunctive therapy for major depressive disorder, including cases resistant to standard treatments (37), indicating its promise for older individuals with altered neurochemical and hormonal states. Although subgroup analyses revealed some influence of age, type of surgery, and anesthesia method, the high degree of heterogeneity (I²=84%) suggests the presence of other important unmeasured confounding factors. The eight articles included in this review used different anxiety assessment tools (e.g., HAMA vs. VAS-A), and the high heterogeneity may be attributed to varying sensitivities of these tools in measuring state versus trait anxiety or different dimensions of anxiety. Future studies should adopt standardized anxiety assessment instruments and consistent dosing regimens to reduce heterogeneity, thereby enabling a more precise evaluation of the efficacy of esketamine.

Following administration, both (S)-ketamine and (R)-ketamine have been shown to stimulate glutamate release, which subsequently boosts the function of α-amino-3-hydroxy-5-methyl-4-isoxazole propionic acid receptors (AMPARs) (38). Increased activation of AMPARs initiates several intracellular signaling cascades, notably promoting the release of brain-derived neurotrophic factor (BDNF) and stimulating the mechanistic target of rapamycin complex 1 (mTORC1) and TrkB signaling pathways (39). One prominent theory suggests that ketamine preferentially blocks GluN2B subunit-containing NMDA receptors, predominantly found on GABAergic interneurons. This action reduces inhibitory control over cortical pyramidal neurons, leading to elevated synaptic glutamate levels. The increased glutamate subsequently stimulates AMPA receptors, initiating intracellular signaling pathways (40, 41). An alternative mechanism suggests that ketamine inhibits extra synaptic NMDA receptors, resulting in dephosphorylation of eukaryotic elongation factor 2 (eEF2). This process alleviates the suppression of brain-derived neurotrophic factor (BDNF) secretion, thereby facilitating the incorporation of GluA1 and GluA2 subunits into the postsynaptic membrane. Consequently, this enhances AMPA receptor signaling and induces synaptic homeostatic scaling (41, 42). Under stressful conditions such as surgical procedures, NMDA receptor function begins to deteriorate (43). Alterations in cognition and mood stem from dysfunctions in the hippocampus and amygdala, which impair their regulation of top-down control by the prefrontal cortex (PFC) (44). Abnormal NMDA receptor function can contribute to the development and maintenance of anxiety symptoms through various pathways. The influx of calcium through NMDARs triggers an intracellular signaling cascade. This cascade not only mediates local, acute functional synaptic plasticity but also induces changes in gene expression, which in turn further modulate synaptic plasticity (45). The “glutamate burst” hypothesis suggests that ketamine’s blockade of NMDARs on GABAergic interneurons disinhibits pyramidal neurons, thereby enhancing glutamatergic transmission. This glutamate surge is proposed to initiate a cascade that includes increased AMPAR activity and the release of BDNF (46). By influencing membrane properties and synaptic activity, PUFAs(Polyunsaturated fatty acids) especially DHA(docosahexanoic acid) and AA(arachidonic acid) regulate NMDA receptor function. Moreover, studies indicate that omega-3 PUFAs can safeguard glutamatergic neurotransmission from stress-induced dysfunction, which may help prevent the emergence of stress-related symptoms. Critically, these effects are facilitated intracellularly by fatty acid-binding proteins (FABPs), which transport PUFAs to their sites of action (47). Growing evidence positions D-serine, which activates synaptic NMDA receptors more strongly than glycine, as the primary co-agonist for these receptors in limbic regions such as the amygdala, medial prefrontal cortex, and hippocampus. These regions are critically involved in fear memory formation and extinction. Therefore, augmenting NMDA receptor function in these circuits represents a potential strategy for promoting the extinction of fear memories that engage higher-order cognitive processing (32).

This meta-analysis is subject to several limitations. One major concern is the restricted diversity of anesthesia techniques used in the analyzed trials, which led to a relatively modest cumulative sample size-especially concerning spinal anesthesia. The limited methodological range may not adequately reflect the broader potential of esketamine in mitigating perioperative anxiety. Therefore, these findings should be interpreted cautiously and confirmed by future research employing more diverse and extensive methodological frameworks. Moreover, all included participants were recruited from China. Chinese patients may exhibit systematic differences in baseline anxiety levels, cultural perceptions of surgery and anesthesia, perioperative care routines, and concomitant medication patterns compared to other regions. The applicability of the results to other demographic groups and medical settings may be limited. The absence of uniform preoperative anxiety screening protocols and the utilization of diverse evaluation instruments further compromise the generalizability of findings. Such variability makes it challenging to draw direct comparisons regarding esketamine’s effectiveness. Consequently, future high-quality randomized controlled trials involving larger, heterogeneous populations and consistent assessment criteria are essential to establish esketamine’s efficacy in managing perioperative anxiety.

## 5 Conclusion

This meta-analysis demonstrates that esketamine administration during the perioperative period can effectively lower anxiety levels. However, its impact on depressive symptoms, sleep quality, and postoperative pain appears minimal. Importantly, compared to the control group, esketamine did not increase the risk of adverse reactions such as postoperative nausea and vomiting (PONV), dizziness, or nightmares. Future investigations should focus on determining the most effective timing-whether preoperative, intraoperative, or postoperative-and appropriate dosing strategies to enhance anxiolytic benefits while minimizing adverse outcomes. Large-scale, multicenter randomized trials are warranted to evaluate dose-response dynamics, identify the lowest effective dose, and establish upper safety thresholds. Moreover, co-administration of preventive agents during surgery may offer additional protection against potential esketamine-related side effects.

## Data Availability

The original contributions presented in the study are included in the article/**Supplementary Material**. Further inquiries can be directed to the corresponding author.
