# Supplementary material for: Efficacy of esketamine on perioperative anxiety in patients receiving anesthesia: a systematic review and meta-analysis of RCTs
Source: Front Psychiatry. 2026 Feb 3;17:1721985. doi: 10.3389/fpsyt.2026.1721985 (PMC12909511; doi:10.3389/fpsyt.2026.1721985)
Supplement: Supplementary file 1 [file Table1.docx]

**Supplementary materials**

**TableS1 The detailed search strategy**

((("Esketamine" [Supplementary Concept]) OR (((((L-Ketamine) OR (Ketamine)) OR (S-Ketamine)) OR (Kataved)) OR (Spravato))) AND (("Anxiety"[Mesh]) OR ((((((Angst) OR (Nervousness)) OR (Hypervigilance)) OR (Social Anxiety)) OR (Social Anxieties)) OR (Anxiousness)))) AND (Random*)

Pubmed-344


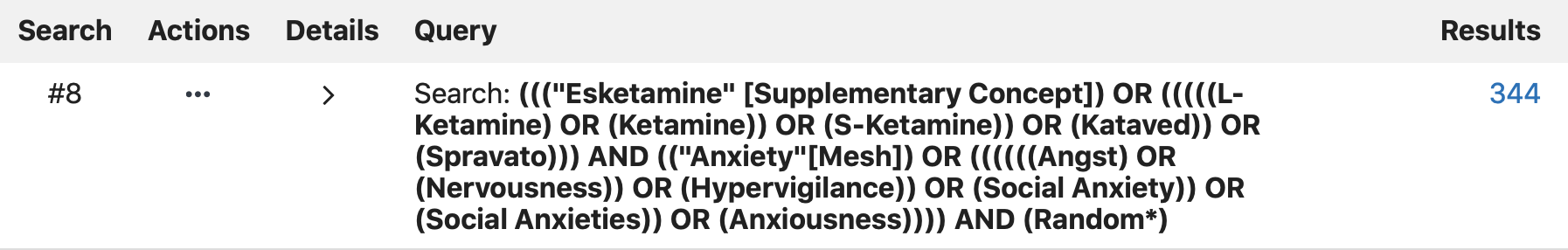


(((Esketamine) OR (((((L-Ketamine) OR (Ketamine)) OR (S-Ketamine)) OR (Kataved)) OR (Spravato))) AND ((Anxiety) OR ((((((Angst) OR (Nervousness)) OR (Hypervigilance)) OR (Social Anxiety)) OR (Social Anxieties)) OR (Anxiousness)))) AND (Random*)

Embase-729


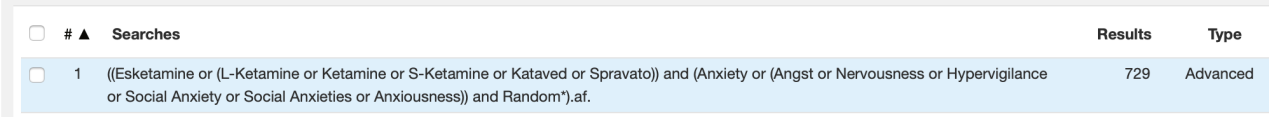


(((Esketamine) OR (((((L-Ketamine) OR (Ketamine)) OR (S-Ketamine)) OR (Kataved)) OR (Spravato))) AND ((Anxiety) OR ((((((Angst) OR (Nervousness)) OR (Hypervigilance)) OR (Social Anxiety)) OR (Social Anxieties)) OR (Anxiousness)))) AND (Random*)

Cochrone-509


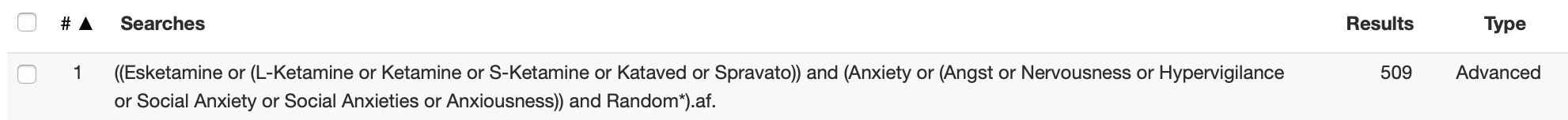


(((Esketamine) OR (((((L-Ketamine) OR (Ketamine)) OR (S-Ketamine)) OR (Kataved)) OR (Spravato))) AND ((Anxiety) OR ((((((Angst) OR (Nervousness)) OR (Hypervigilance)) OR (Social Anxiety)) OR (Social Anxieties)) OR (Anxiousness)))) AND (Random*)

WOS-344


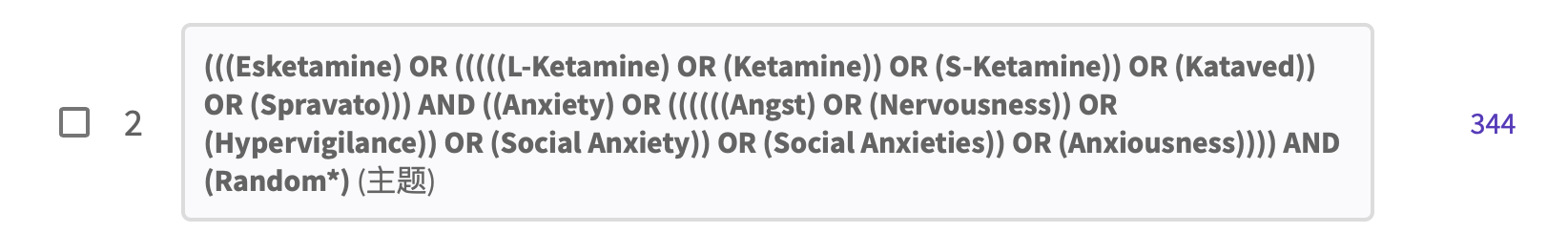


(((Esketamine) OR (((((L-Ketamine) OR (Ketamine)) OR (S-Ketamine)) OR (Kataved)) OR (Spravato))) AND ((Anxiety) OR ((((((Angst) OR (Nervousness)) OR (Hypervigilance)) OR (Social Anxiety)) OR (Social Anxieties)) OR (Anxiousness)))) AND (Random*) (topic)


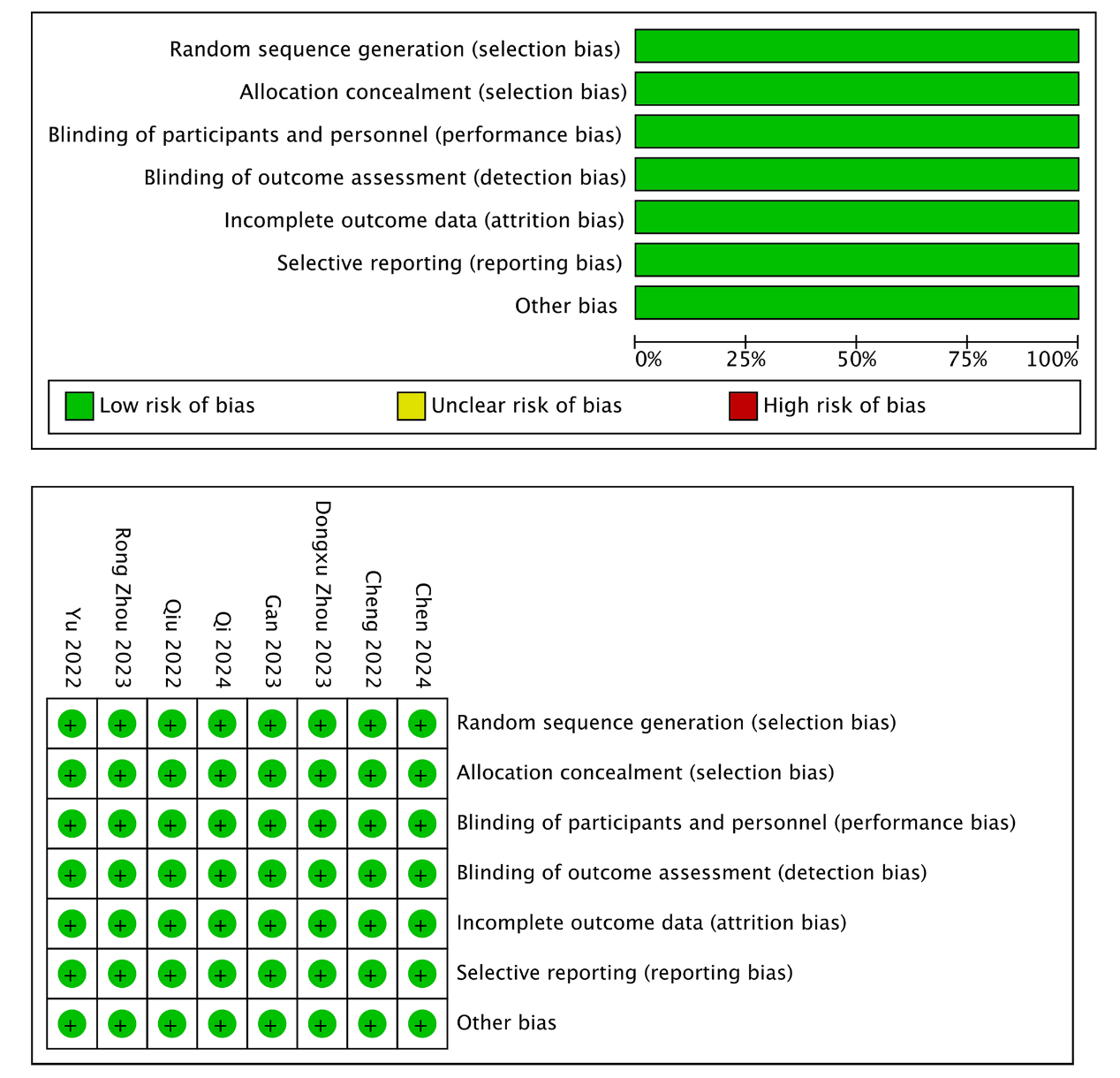


**Figure S1 Quality assessment of all eligible studie.**
